# Exploring Heart Rate Variability Changes Following Respiratory Training in Individuals with Panic Disorder

**DOI:** 10.1192/j.eurpsy.2025.2113

**Published:** 2025-08-26

**Authors:** S. Daccò, D. Caldirola, A. Alciati, A. Spiti, G. Perna

**Affiliations:** 1 Department of Biomedical Sciences, Humanitas University, Pieve Emanuele; 2 Humanitas San Pio X Hospital, Milan; 3 IRCCS Humanitas Research Hospital, Rozzano, Italy

## Abstract

**Introduction:**

Studies reported an association between panic disorder (PD) and diminished heart rate variability (HRV) (Zhang *et al.* J Affect Disord 2020; 15, 267 297-306), potentially heightening their susceptibility to cardiovascular mortality and diminished quality of life. Preliminary research has demonstrated that HRV-biofeedback training can enhance the regulation of autonomic function in both healthy individuals (Schumann *et al.* Front Neurosci 202;15 691988) and patients with anxiety (Herhaus *et al.* Psychosom Med 2022; 84, 2 199-209).

**Objectives:**

We examined the impact of HRV-biofeedback training intervention on autonomic activity modulation among outpatients with PD with or without agoraphobia (AGO).

**Methods:**

We conducted a retrospective observational pilot study including 10 outpatients (five females and five males; median age = 35.5 years) diagnosed with PD (9 with comorbid AGO and 1 without). The clinician-administered Panic Associated Symptoms Scale (PASS) was used to assess PD and AGO severity. All the included patients underwent a physiological assessment to determine HRV parameters and breathing rate during a 5-minute resting condition at baseline (T0) and follow-up (T1) assessment. Between T0 and T1 assessments, all patients underwent HRV-biofeedback training. Pre-post comparisons were conducted using the Wilcoxon Signed Ranks Test. To evaluate the impact of psychotropic medications, we used the Mann-Whitney U test to compare HRV parameters and disorder severity between patients with and without medications, at T0 and T1. Significance level was set at p<0.05.

**Results:**

HRV-biofeedback training among individuals with PD with or without AGO fostered notable enhancements in RMSSD (Z = -2.08; p < 0.01), SDNN (Z = -2.5; p = 0.01), and LF (Z = -2.09; p = 0.04) parameters at T1 compared to T0. Furthermore, a significant decrease in heart rate (Z = -2.19; p = 0.03) was shown. These enhancements didn’t seem related to psychopharmacological medications and disorder severity.

**Conclusions:**

Despite limitations (e.g., small sample, no control group, lack of comorbidity and T1-PD severity assessment, missing inclusion/exclusion criteria, and unaccounted confounding variables affecting HRV), our study suggests that HRV biofeedback training may improve autonomic regulation in PD patients, aligning with existing literature (Herhaus *et al.* Psychosom Med 2022; 84, 2 199-209). Further research is needed to determine to what extent bio-feedback training can help to reduce panic symptoms and disorder severity.

**Disclosure of Interest:**

None Declared

